# Native Listeners’ Use of Information in Parsing Ambiguous Casual Speech

**DOI:** 10.3390/brainsci12070930

**Published:** 2022-07-15

**Authors:** Natasha Warner, Dan Brenner, Benjamin V. Tucker, Mirjam Ernestus

**Affiliations:** 1 Department of Linguistics, University of Arizona, Tucson, AZ 85721, USA; wobaidan@gmail.com; 2 Department of Linguistics, University of Alberta, Edmonton, AB T6G 2E7, Canada; benjamin.tucker@ualberta.ca; 3 Centre for Language Studies, Radboud University, 6500 HD Nijmegen, The Netherlands; mirjam.ernestus@ru.nl

**Keywords:** reduced speech, conversation, comprehension, context, acoustic cues

## Abstract

In conversational speech, phones and entire syllables are often missing. This can make “he’s” and “he was” homophonous, realized for example as [ɨz]. Similarly, “you’re” and “you were” can both be realized as [jɚ], [ɨ], etc. We investigated what types of information native listeners use to perceive such verb tenses. Possible types included acoustic cues in the phrase (e.g., in “he was”), the rate of the surrounding speech, and syntactic and semantic information in the utterance, such as the presence of time adverbs such as “yesterday” or other tensed verbs. We extracted utterances such as “So they’re gonna have like a random roommate” and “And he was like, ‘What’s wrong?!’” from recordings of spontaneous conversations. We presented parts of these utterances to listeners, in either a written or auditory modality, to determine which types of information facilitated listeners’ comprehension. Listeners rely primarily on acoustic cues in or near the target words rather than meaning and syntactic information in the context. While that information also improves comprehension in some conditions, the acoustic cues in the target itself are strong enough to reverse the percept that listeners gain from all other information together. Acoustic cues override other information in comprehending reduced productions in conversational speech.

## 1. Introduction

In normal daily-life conversations, humans convey information to each other efficiently, but do not produce all of the phones that they would in a careful speech version of the same sentences (although some of those phones may leave traces through coarticulation) [1,2,3]. This paper investigates what sources of information listeners use to understand reduced speech. For example, among normal, casual conversations we recorded, we have found tokens where speakers pronounced “gonna have to” as [gɔ̰ʔtə̰], or “a little” as a sonorant stretch of the waveform with low F2 and some change in formants and amplitude, but no distinguishable segments. (Audio examples are available at http://nwarner.faculty.arizona.edu/content/6 accessed on 13 July 2022) Such reduced speech clearly does not contain the same perceptual cues as a careful speech production does. Still, listeners usually perceive casual conversational speech with little difficulty, at least if they hear it in context and the speech is in their native language.

If listeners hear reduced speech out of the context it was produced in, they typically do not perceive the words of the reduced speech accurately. Koopmans-van Beinum [4] showed that listeners are very inaccurate at perceiving individual vowels that have been extracted from spontaneous speech. Ernestus et al. [5] showed that even very common words such as Dutch “mogelijk” /moxələk/ ‘possibly’ are recognized quite poorly (approximately 50% correct word identification) when highly reduced pronunciations such as [mok] are presented in isolation, extracted from the original context. Janse and Ernestus [6] further found that contextual information is more helpful to listeners if it is presented auditorily rather than orthographically. Arai [7], working on Japanese, similarly found that listeners badly misperceive strings of reduced speech out of context, for example hearing a recording of the five-mora string /kuiebaho/ as only the two morae /kebo/. These studies also confirmed that listeners perceive the same speech accurately when they hear it in context. Saerens et al. [8] investigated the perception of French voiceless stops with ambiguous voicing excised from conversational speech. They found that the lexical context of the rest of the words in the sentence helps listeners recover the intended voicing of the stops, but that secondary acoustic cues in or near the stop also play a role.

In some cases, reduction not only alters perceptual cues or segments, but it also creates ambiguity about what word was intended. In reduced speech, “he’s” and “he was” can sound homophonous, with some of the reduced tokens sounding like [hɨz], and some so reduced that the only trace of the word(s) in the waveform is a [z] or [s]. (Audio examples are available on the website mentioned above.) Similarly, “we’re” and “we were” can both sound like [wɚ] or possibly just [ɚ]. In both of these cases (“he’s/he was” and “we’re/we were”), reduction obscures the distinction between present and past tense, similar to how reduction and contextual speech rate can obscure the singular/plural distinction in sentences such as “The Petersons are looking to buy a brown hen/brown hens soon” [9]. Thus, reduction may not only inhibit how quickly and accurately listeners recognize content words in speech [10,11], it may also make function words with different meanings like “he’s” and “he was” homophonous. Because function words are important for parsing the structure and meaning of the sentence, this makes reduction especially relevant for listeners’ syntactic processing.

The speech signal includes several types of information that listeners might use to parse reduced speech and disambiguate reduced function words. One common assumption is that listeners perceive the other words of the utterance, and they retrieve the intended meaning through the syntactic or semantic context. For example, if one hears a highly reduced token of “We were supposed to see it yesterday” in which “we were” sounds like “we’re,” the word “yesterday” will allow the listener to realize the verb is past tense. This would likely be true even if the word “yesterday” is also reduced, since “yesterday” is more distinct from other lexical entries than “we’re” and “we were” are from each other. When the authors play examples of reduced speech in classes or at conferences and ask the audience how they think listeners might be able to understand the speech, the first answer given is consistently “context,” and when they are pressed to explain what they mean, they give some version of this explanation.

Several other types of information are also present in the signal. Most obviously, there is acoustic information in the reduced speech. It may not be sufficient for listeners to recognize the words, and it may even provide misleading perceptual cues. For example, our highly reduced recording of “gonna have to” mentioned above contains a creaky voice through a large part of the voicing, which may suggest a glottal stop. We find that listeners often misperceive this string as “got to,” where a glottal stop is likely. If a token of “he was” out of context sounds to listeners like “he’s,” this means that the perceptual cues that are present are misleading. Any stretch of speech, no matter how reduced, contains acoustic information and hence perceptual cues, whether to the words the speaker intended or to something else.

Another type of information listeners use is speech rate. Listeners use speech rate within the same syllable to adjust the boundaries between aspirated and unaspirated stop categories [12,13]. Listeners use the speech rate of the surrounding utterance to help distinguish vowels such as /ɪ/ vs. /i/ (where /ɪ/ is intrinsically shorter), accepting a longer vowel as /ɪ/ if surrounding speech rate is slow [14]. At the word level, in phrases such as “leisure (or) time,” altering the speech rate of either the surrounding context or the function word “or” can determine whether listeners perceive the function word at all [15]. If the function word is shorter than would be expected for the surrounding speech rate, either because the “or” was shortened or the surrounding speech was lengthened, listeners perceive “leisure time” instead of “leisure or time.” Conversely, if the /r/ portion of the signal “leisure” is long relative to the surrounding speech rate, listeners may perceive an “or” that the speaker did not produce. Niebuhr and Kohler [16] showed a related result for German. This shows that listeners use the speech rate of the context to determine whether acoustic cues last long enough to constitute additional segments or words. Brown et al. [9] showed this effect through eye-tracking, establishing that it occurs in real time as part of how listeners develop expectations about upcoming words. Heffner et al. [17] investigated how listeners combine the information about context speech rate with acoustic cues within the word.

In the current work, if listeners hear [hɨz] with a relatively long duration, but the surrounding speech is very fast, they may hear “he was” instead of “he’s.” That is, the boundary between what counts as a good token of “he’s” vs. a good token of “he was” may depend on duration, adjusted for the surrounding speech rate. One can imagine this as a subconscious process of “That was too long to be just ‘he’s’ considering how fast this speech is going. Something must have been deleted. Maybe the speaker said ‘he was.’”

In addition to syntactic and semantic information from other words in the utterance, listeners engaged in a conversation may also benefit from discourse information in the larger context, beyond the utterance. Knowing that one’s interlocutor is discussing wedding plans, her part-time job, or a relative’s health decisions may help the listener to adjust expectations for likeliness of words. However, this is probably less helpful for strings such as “he’s” vs. “he was,” which could both occur in most conversations. Another type of information available to listeners is information about a speaker’s voice, both about properties such as vocal-tract conditioned vowel space (e.g., [18]) or the degree of habitual nasalization, and about idiosyncrasies or dialectal features [19]. Exposure to a longer sample of a speaker’s voice allows listeners to adjust their expectations for the speaker’s typical pronunciation. Furthermore, Brouwer et al. [20] found that if listeners have been hearing reduced spontaneous speech preceding a target, they penalize acoustic mismatches with lexical entries less strongly, so the speech style of the context also supplies information.

Van de Ven et al. [21] investigated how well semantically related words prime word recognition if the primes or targets are reduced vs. carefully pronounced. They found that the semantic information in reduced pronunciations of words does not help listeners to recognize subsequent words unless listeners are given more time than usual to fully process the reduced words before the related word is presented. This study used a priming methodology with words presented in isolation, so it did not test whether listeners use semantic information in the preceding parts of the sentence or discourse, but instead whether the activation of a related semantic concept outside of a discourse helps with isolated word recognition.

Using a different method, van de Ven et al. [22] showed that native speakers are able to use the syntactic and semantic information in a surrounding sentence to help them predict a missing adverb in the sentence at better than chance, but still low, rates. Native Dutch speakers in their experiment were able to predict the missing word “altijd” ‘always’ at better-than-chance rates in a sentence such as “Ik vertrouw altijd maar op mijn goede geluk” ‘I always rely on my good luck.’ The success rate at predicting such words was higher than would be expected based on n-gram probabilities, and was higher when listeners were able to hear the surrounding context auditorily rather than reading it. This indicates that there is some information in the phonetics, syntax, and semantics of the context, even for adverbs that are not predictable in the sentence. However, van de Ven et al. [22] also found that listeners obtain far more information about the words from hearing the word itself than from context. Van de Ven and Ernestus [23] presented various portions of the speech signal around and during reduced words in Dutch conversational speech, and found that listeners make less use of bigram probability based on the preceding or following word as they are given more acoustic cues from the target word to work with. Drijvers et al. [24] studied listeners’ neuronal oscillations while hearing reduced vs. clearly pronounced word forms in various contexts, and found that reduced forms impose a higher cognitive load during recognition, which prevents lexical activation from spreading through the semantic network as quickly when words are reduced.

In order to determine which types of information (e.g., acoustic, speech rate, syntax/semantics) help listeners disambiguate forms such as “he’s” vs. “he was” or “we’re” vs. “we were” in casual, reduced speech, we conducted a series of experiments. We extracted utterances containing words/phrases such as “he’s,” “we were,” “she was” etc., from recordings of spontaneous casual conversations that had been made for another experiment [25]. Some examples are “’Cuz he already told Steve he was in the wedding” or “When we were outside the bookstore...” (Underline indicates the target word/phrase.) A few items had a word other than a pronoun as the first word of the target, but these had the same potential tense ambiguity (e.g., “Katie was/Katie’s”). We presented stimuli based on these utterances to participants with various types of context or information available, in order to determine how well listeners could disambiguate the reduced speech if given access to some types of information but not others. Experiments 1 and 2 provide baseline measures of how much information is available from the syntax and semantics of surrounding words, without the target words themselves. Experiment 3 turns to perception of the target word/phrase.

## 2. Experiment 1: Syntactic and Semantic Context without Acoustics (Orthographic Presentation)

Since the utterances we use as stimuli were taken from spontaneous, natural conversations, and were not constructed to be either semantically predictable or not, we need to establish a baseline of how much information about the target phrase/word one can gain just from the syntax and semantics of the rest of the utterance. In Experiment 1, we presented the utterances to participants written on a computer screen, with a blank for the target word/phrase, and asked participants to choose whether the present or past version of the target would be more likely to appear in the blank in the utterance. For example, participants would see “’Cuz he already told Steve ____ in the wedding” on the computer screen, with “he’s/he is” and “he was” printed below. Participants pressed a button on a response box to indicate which alternative they thought was more likely to fill in the blank. Thus, in this experiment, participants had access to all the syntactic and semantic information in the surrounding context, but did not have access to any acoustic information, either about the target or about the context.

### 2.1. Methods

#### 2.1.1. Materials

A total of 184 utterances containing words/phrases such as “he is,” “he’s,” “she was,” “she’s,” “we’re,” “we were,” were chosen from recordings of 18 native speakers of American English who were originally recorded for a production study of spontaneous conversational speech (a superset of the speakers in [25]) (The study in [25] involved labor-intensive acoustic labeling that precluded measuring all of the participants who volunteered and were recorded at that time). Sample items appear in Appendix A (Table A1). Most of the speakers were completely monolingual in English until at least their teenage years, when they began taking language classes in school. Some had limited exposure to another language (e.g., Spanish, a Chinese language, Canadian French) in the home as children, but all were strongly English dominant and grew up in the U.S. The speakers were undergraduate students at the University of Arizona at the time the recordings were made (2005), and most were from the Southwestern U.S. or California. None spoke a dialect that was notably different from varieties typically heard in Arizona.

Speakers sat in a sound-protected booth and wore a high quality head-mounted microphone over the opposite ear from the one where they habitually held a telephone. Each speaker called a friend or family member and held a conversation of approximately 10 min on whatever topics they wished to discuss. Further details of the methods for obtaining this speech are available in [25]. Speech was recorded through the microphone, not the telephone. The telephone was only used to allow a casual conversation with a well-known interlocutor, while still recording in a sound booth. This method succeeded in eliciting highly informal, casual speech, as shown by the range of topics discussed (including fraternities and drinking games as well as courses, part-time jobs, and family members).

During past work with these recordings, research assistants from a similar background to that of the speakers (undergraduate students at the same university a few years after the recordings were made) produced orthographic transcriptions of the recordings. We used these to locate sufficient numbers of tokens containing the strings “X is” (either contracted or not; both “he is” and “he’s” were included), “X was,” “X are” (including contracted forms, hence both “we’re” and “we are”), and “X were.” In each case, the longer past tense form had to be reducible to be homophonous with the shorter present tense form. For example, “Grammy’s” could be used, because the words “Grammy was” could potentially be reduced to sound similar to “Grammy’s,” but “I was” could not be used, as there is no related shorter form “I’se” in English as spoken in Arizona with which it could become homophonous. All but 19 items had a personal pronoun (e.g., “he, she, it, we, they”) in the X position. The remaining 19 included 7 items with “there,” three with “who,” two with “how,” and one each of “parents, weekend, Katie, what, so, Grammy, everybody.” The average number of words in the utterance before the target item was 3.08, and the average number after it was 4.98. The number of items drawn from each speaker’s recording ranged from 2 to 42, and depended on how often the speaker used the target phrases. Using stimuli drawn from spontaneous conversation means that the stimuli are quite variable, for example in the sentence structure and focus in or near the target word/phrase. However, it has the advantage that the speech participants respond to is representative of what they hear in informal conversations in daily life, reducing the chance of task effects.

Each item was checked by the first author, as well as having been identified from the longer recording based on transcriptions made by the research assistants, whose age and dialect was a good match to the speakers’. Thus, each item was checked to determine whether the particular token was produced with “is” or “was” by at least two native speakers of American English who heard the entire discourse context of the longer recording and could listen to the utterance and any amount of context as many times as they wished. The first author agreed with the research assistants’ perception of all items that were used. The orthographic transcriptions of these items form the materials for Experiment 1.

#### 2.1.2. Participants

46 native speakers of English participated in Experiment 1. All were students in introductory Linguistics courses at the University of Arizona, and all had either been monolingual in American English until at least puberty, or had had some exposure to another language (e.g., German, Korean, Marathi, Gujerati, Spanish) in the home but were strongly English-dominant. All had grown up entirely in the U.S. Participants received extra credit in their Linguistics course as compensation.

#### 2.1.3. Procedures

Participants sat in a sound-protected booth with a computer monitor outside the window of the booth. Participants saw each item in written form on the computer monitor, with a blank inserted for the target word/phrase, e.g., “’Cuz he already told Steve ____ in the wedding.” Below the utterance the response options were printed, giving the present and past tense options, adjusted to use the correct word before the verb. That is, for this item the response options were “he’s/he is” and “he was,” while for the stimulus “And ____ huge houses too, it was weird, like” (“they’re” deleted), the response options were “they’re/they are” and “they were.” The response options did not distinguish between contracted vs. full forms of present tense: participants were only asked to choose between present and past forms, not between “he is” vs. “he’s,” for example. Since for this experiment, participants did not hear the target or the context, they were instructed to choose which of the two response alternatives they thought would be more likely to occur in the blank. Participants were instructed that the sentences came from casual conversations.

The EPrime software (Psychology Software Tools [26]) was used to present stimuli and record responses. Participants pressed buttons on a response box to indicate whether they chose the left or right response on the monitor. The two response alternatives were randomly assigned to left and right position. Participants first responded to 6 practice items, followed by the full list of stimuli, in a different random order for each participant. After each stimulus appeared on the screen, the participant had up to 10 s to read it and respond, after which the program advanced to the next stimulus. On 1.2% of trials, participants failed to respond. The average reaction time was slightly over 4 s, reflecting time to read the stimulus. Six participants were mistakenly presented with a version of the experiment that omitted three items.

Approximately 90 additional items were included. Most were for an additional distinction (“X him” vs. “X them,” as in “got ‘em”), and some were additional items for the current conditions. These will not be discussed further, because the length of the subsequent experiments precluded the use of these additional items in the other experiments. The entire experiment, including the additional items, took approximately 35 min.

### 2.2. Results

**Figure 1:**
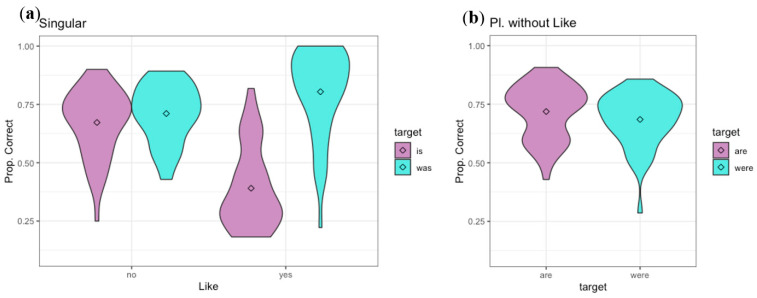
Results with orthographic presentation (distribution of listeners’ averages over items). Dots indicate means for each condition. (**a**) “Is” and “was” targets. (**b**) “Are” and “were” targets.

Results for Experiment 1, as proportion correct, appear in Figure 1. (Using proportion correct as the dependent variable, instead of proportion present tense responses (or proportion past), focuses the analysis on investigating bias rather than whether listeners can distinguish the present from past. The d’ analysis below focuses on the latter question.) During analysis, it became clear that participants’ responses differed strongly depending on whether the target is followed by the quotative or discourse particle “like” or not (e.g., the stimuli “She’s like, ‘No! No more laptops!’”, “And he was like, ‘What’s wrong?!’”, and “Yeah he was like, ignoring me until he right, he, ‘til right before he got on the bus.”). This could be because speakers have the option of using the historical present to report a past conversation or situation, as in the first of these examples, making verb tense relatively uninformative before “like.” Therefore, the presence of the word “like” after the target was included as a post hoc factor, as in Figure 1. Past research on “like” usage [27,28,29,30] suggests that these constructions may have properties that other usages of “he was, we were” etc. do not, confirming the need to include presence of following “like” as a factor. We did not attempt to distinguish among usages of “like,” since it can be difficult to determine whether a given usage is quotative or not, and some stimuli ended with the “like” because the speaker made a long pause or stopped the utterance (e.g., “So like, she’s like…”).

“Like” occurs less often after plural subjects than singular subjects in our speakers’ conversations (contrary to [27], perhaps suggesting a change in the intervening 15 years). The stimuli contained only two items with the target verb “are” followed by “like” and only seven with “were” followed by “like.” This is too few items to provide reliable data, so we chose not to analyze the few items with “X are/were like” statistically. Since the singular conditions (“is, was”) show a very strong difference in behavior depending on the presence of “like,” we therefore analyzed the data in three subsets: “is” vs. “was” targets without a following “like,” “is” vs. “was” targets with a following “like,” and “are” vs. “were” targets without a following “like.” Each analysis had the intended tense of the verb (present, past) as the fixed factor. (One could also analyze either both sets of “is” vs. “was” targets, or both sets of targets without “like,” in a larger analysis with an additional factor. When tested, this type of higher-order analysis revealed an interaction that motivated testing each subset separately. (The higher-order LMEs generally showed significant interactions but also had failure to converge or singular fit warnings, and so we do not report numerical details of those models. However, to further motivate testing subsets of the data (simple effects tests) based on significant interactions, we performed by-subject ANOVAs (hence averaged over items). Because of the absence of plural “like” items, we performed an analysis on all of the singular data (with “like” and tense as factors). Both factors are within-subjects. A significant interaction motivated testing simple effects of tense of stimulus. Singular data: tense: F(1,45) = 49.00, like: F(1,45) = 44.93, interaction: F(1,45) = 110.34, all *p*’s < 0.001. One could also analyze all of the data without “like” (with tense and number as factors), but the interaction in the singular data and absence of plural-like data already motivate testing simple effects of tense.).

We analyzed the data using generalized linear mixed effects models with a binomial link function as implemented in the lme4 package ([31] version 1.1–29) of R (using glmer), with the correctness of response as the dependent variable. The fixed factor tested for each present/past pair was the tense of the target word/phrase as produced by the speaker in each item (reference level: past). (For example, a participant sees “’Cuz he already told Steve ___ in the wedding,” which was originally produced with “he was.” The participant has a choice of “he’s/he is” and “he was” as response options. If the participant selects “he’s/he is” this is scored as incorrect, while selecting “he was” is scored as correct. The correctness of response is evaluated relative to what was originally produced in the target word/phrase, regardless of whether any other tensed verbs appear in the utterance. The independent variable of tense in the statistical analysis allows an analysis of how participants’ accuracy on stimuli that originally contained present might differ from those that originally contained past.) (Models including larger subsets of the data at once, and more factors, showed significant interactions, so the tense factor had to be tested for each set separately.) Model selection was performed using an ANOVA comparison. Random intercepts for subject (participant) and item (sentence), as well as random slopes by subject for the tense factor, were included if the model converged and did not give singular fit warnings. Random intercepts for speaker (who produced the stimulus) were also tested and found not to improve the model’s fit. Since participants did not hear the voices that produced the stimuli in this experiment, it is not surprising that the speaker random intercepts did not improve the models. (All three subsets (*is/was without like, is/was with like,* and *are/were without like*) used the model Correct ~ Tense + (1+Tense|Subject) + (1|Item).)

The model for the “is/was” data not followed by “like” showed no significant effect of the tense of the stimulus (β = −0.21, z = −0.78, *p* = 0.44). The model for the “is/was like” data showed significantly more correct responses for “was” than “is” (β = −2.63, z = −6.06, *p* < 0.001). The model for the “are/were” data without “like” also showed no effect of tense (β = 0.16, z = 0.62, *p* = 0.54). The significance of the tense effect in the “is/was like” data does not indicate that “was like” is necessarily easier to perceive than “is like,” but rather that participants are biased toward the “was” response. This could be because the quotative “like” is used to introduce reported speech, which must necessarily have been uttered in the past. Although one can use historical present to describe past speech with “he’s like,...,” participants seem to assume that speech uttered in the past will be reported in the past, and favor the “was” response. This leads to a high accuracy when the stimulus actually contains “was” and a low accuracy when it actually contains “is.”

To further evaluate bias, we examined the average accuracy across past and present tense items, the detectability of the past/present distinction (d’), and bias (β) for each present/past verb pair (Table 1) (The corresponding results for later experiments of the paper are presented as well, and will be discussed below). The average accuracy, 58.3–70.2% for the various conditions, is substantially above chance. The d’ value for both pairs without following “like,” at slightly more than one, indicates that listeners were able to extract some information about whether the verb was more likely to be present or past tense, but they were still far from being able to accurately recover tense. For the “is/was like” pair, the d’ is only 0.504, showing that this context offers only very weak information about which verb tense was intended. The value for bias confirms that participants were biased toward the past response for this pair.

### 2.3. Discussion

The results show that native speakers of English evaluate verb tense differently in phrases with a following “like” than in phrases without. In both cases, on average across all sentences used, they were able to extract at least some information about whether the verb is more likely to be present or past tense from the surrounding syntactic and semantic context. The sentential context in the sentences without “like” conveys more information about verb tense (69% correct) than in those with “like” (58% correct), as indicated by the higher d’. This is not surprising, since speakers have the option of using historical present to report past speech or events using the quotative “like.” The bias toward “was” in this condition suggests that participants did not usually take the historical present option into account in reading the sentences. Instead, they seem to have assumed that a verb reporting past speech would be in the past tense, thus favoring the “was” response.

In the conditions without a following “like,” participants had almost no bias, favoring neither present nor past verbs. Most of the items without “like” contain clearer syntactic or semantic information, as in “And so they were getting back to Desert [a school] right when Eric and I got there” or “’Cuz I can’t hang out with anyone, ‘cuz they’re, everyone’s gonna be studying.” In both of these utterances, the opposite tense would be very unlikely. However, not all items without “like” contain tense information outside the target phrase itself, as in “But she’s bored out of her mind,” which could use either tense.

These results show how well native English speakers are able to recover the tense of the verb based solely on syntactic and semantic information. The average correct response rate of 69% across all stimuli without “like,” and the d’ for these conditions of approximately one, show that participants are able to recover some information from the sentential context, but not enough to determine the verb tense with consistent accuracy. The two alternative forced choice task with an orthographic presentation gives a clear estimate of how much information is available in the syntax and semantics of the context of these particular utterances, without including any acoustic information, information from coarticulation with the target words, or any other auditory source. This provides a baseline for comparison with Experiments 2 and 3. It is possible that participants evaluate the verb tense differently than they would if they had heard the stimuli, even though they have been told that the material comes from conversations. Experiment 2 investigated how much information listeners can extract from syntactic, semantic, and prosodic context through the auditory modality, in order to provide an alternative baseline measure of how much information is available in the context.

## 3. Experiment 2: Syntactic and Semantic Context with Auditory Information

Experiment 2 replicated Experiment 1, but in the auditory modality. Instead of the target word/phrase being represented by a written blank, the portion of the speech signal corresponding to it was replaced with a beep sound similar to a square wave. The duration of the beep was standardized for all stimuli, so that duration of the target could not serve as a perceptual cue and was not confusing. Thus, listeners in this experiment had access to all of the information that participants in Experiment 1 did, plus the prosodic information in the rest of the utterance. Hence, they had access to all of the information except that of the target itself. Crucially, listeners in Experiment 2 still had no access to any acoustic cues during the target word/phrase itself. Using the auditory presentation modality may make the casual, conversational nature of the utterances more obvious to listeners. This task may also impose a higher processing load and may lead to more error and a less effective use of the information that is available, simply because the speech in most of the stimuli is fast and is presented just once, whereas participants in Experiment 1 could re-read the stimuli if they wished. Thus, we made no specific prediction about whether listeners could extract more information from the utterance context in Experiment 1 or Experiment 2, since Experiment 2 contains somewhat more information (prosody), but it is also a more difficult task.

### 3.1. Methods

#### 3.1.1. Materials

The materials were made from the original conversational recordings of the 184 items of Experiment 1. Each item was extracted from the conversation from which it was recorded. The portion corresponding to the target word/phrase (e.g., “he’s, she was, we were,” etc.) was located and removed, and replaced by 262 ms of a beep sound. (This sound was a periodic wave with harmonics that are odd-numbered multiples of the fundamental frequency, approximating a square wave.) The 262 ms duration was the average duration of all the target portions. The boundaries for the portion to replace with a beep were adjusted to the nearest zero-crossing to avoid introducing artifacts.

Criteria for placing the boundaries at the edge of the items, and at the edge of the target word/phrase to be replaced by the beep, depended on the voicing and manner of the sounds at the boundaries and on how the sounds were realized phonetically in the particular token. All the boundaries were placed manually by inspecting the waveform and spectrogram. The complete item often began or ended at a pause, in which case the boundary between silence and voicing (for all voiced segments), frication noise, or bursts was identified as the edge of the item. When the item did not begin or end at a pause, the boundary criteria were the same as for the boundaries around the target word/phrase, described below.

For locating the boundaries of the target word/phrase (e.g., the outer edges of “he’s,” “you were”), the boundary between a vowel or sonorant and the preceding voiceless obstruent was placed at the onset of voicing. For example, in “I guess he was on the phone,” (target underlined) there was strong frication noise for the /s/ of “guess” and no change in the frequency of the noise that would indicate an [h]. The ”h” of “he was” was absent in this token. Therefore, the boundary between “guess” and the target “he was” was placed at onset of voicing. Because voicing frequently continues well into the closure of a post-vocalic voiceless stop, the boundary between a vowel or sonorant consonant and a following voiceless obstruent phoneme was placed at offset of F2, rather than the cessation of voicing (e.g., “you were telling me”). Boundaries between a vowel and a voiced obstruent were placed at the onset/offset of F2 (for example in “he was on the phone,” with the /z/ fully voiced, the boundary between “he was” and “on” was placed at onset of F2). For boundaries between a vowel and a nasal, the boundary was placed at the sudden change in frequency distribution of energy visible in the spectrogram. For a voiceless stop burst with a following fricative (as in “like he’s”), the boundary was placed at the change in quality of frication noise from broadband burst noise to frication noise.

**Figure 2:**
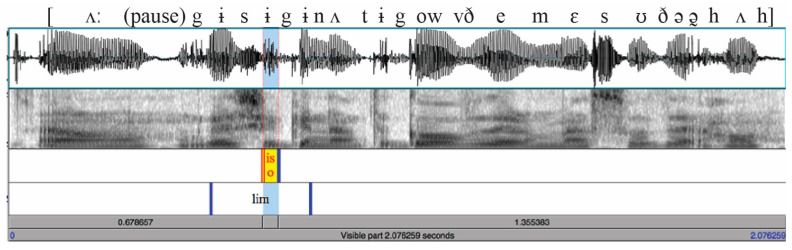
Waveform and spectrogram of a stimulus “Oh, guess you’re gonna hafta go over there and mess with it, huh?” (referring to repairing a computer), containing highly reduced speech, with the target “you’re” realized as a single central vowel. The portion marked “iso” is the portion corresponding to the target “you’re,” and was replaced by a beep in Experiment 2. The portions “iso” and “lim” are explained for Experiment 3 below.

In these spontaneous speech recordings, many sounds one would normally expect to find in the words were not present. For example, in one stimulus containing “and you’re gonna,” this string was realized as [ɪ̃:jɚɰɨ̃nə̃], with the “and” reduced to a nasalized vowel assimilated to the following “j”, and the following ”g” was realized as a weak velar glide. Boundaries between a vowel and a glide, whether these were the expected segments or a result of reduction as in “you’re gonna” here, were placed at the most sudden change in amplitude of formants for the glide, or if there was no change in formant amplitude, at the most sudden acoustic change of any sort visible in the spectrogram. If no acoustic change was present at all (as in the boundary between “and you’re” [ɪ̃:jɚ] in this case), then the boundary was placed in the middle of that sound. This was also the case if the first/last segment of the target was adjacent to another instance of the same phoneme, as in “they’re recording.” Boundary placement was based on the phonetic realization of the particular production, not on what segments would be expected. For example, in “guess you’re gonna” realized as [gɨsɨgɨnʌ] (Figure 2), with a central vowel as the only realization of “you’re”, the boundaries for “you’re” were placed at onset of voicing for the “s”-vowel boundary and the offset of F2 for the vowel-“g” boundary. Because the placement of such boundaries can be difficult in spontaneous speech, all boundaries were also verified auditorily to make sure that segments of adjacent words were not included within the target portion, so that the target portion itself would not contain excessive cues to its neighboring words. The placement of boundaries was conducted by hand labeling, using Praat [32].

#### 3.1.2. Participants

For this experiment, 111 native speakers of American English participated. They were drawn from the same population as the participants in Experiment 1 in a different semester and did not participate in Experiment 1. (The higher number of participants reflects solely the larger number of students wishing to participate at that time.) As in Experiment 1, all participants were either monolingual in English until at least puberty, or had received some exposure to another language in the home but were English-dominant and had grown up in the U.S. No listeners reported any speech or hearing problems.

#### 3.1.3. Procedures

Listeners were seated in a sound-protected booth and heard the stimuli over headphones. The E-Prime software was used to present stimuli and record responses. Listeners first heard 5 practice items similar to the test items, and then heard the 184 test items (and an additional 3 items that were later eliminated for comparability across experiments). The stimuli were blocked by speaker, so that listeners would be able to adjust to the phonetic features of a given speaker, as happens in the perception of conversation in daily life. At the beginning of each speaker block, a filler item by the same speaker was inserted to give listeners a chance to adjust to the new voice before data were collected. These acclimation items were not indicated to the listeners in any way, and listeners responded to them just as for test items. Each listener received the speaker blocks and the items within each speaker block in a different random order. Each stimulus was presented only once.

For each item, the listener heard the entire utterance with the target word/phrase replaced by a beep, as described above (e.g., “’Cuz he already told Steve [beep] in the wedding”). The response options were the same as for Experiment 1, but the utterances themselves were not orthographically displayed on the monitor, only the response options were (e.g., “he’s/he is” and “he was” or “they’re/they are” and “they were” and so on as appropriate to the item were displayed on the screen). Items were randomly assigned to have the correct response appear on the left vs. the right side of the screen. Listeners responded by means of the E-Prime response box, as in Experiment 1. If the listener did not respond, the program advanced to the next stimulus 9 s after onset of the stimulus; this occurred for 352 trials (1.7% of trials were excluded from the data below). The median length of time listeners took to respond on all other trials was approximately 3 s from the onset of the stimulus. The experiment took approximately 25 min. This task was difficult. Anecdotally, when hearing these fast and casual stimuli, it was often difficult even to be sure where in the sentence the rather short beep occurred.

### 3.2. Results

**Figure 3:**
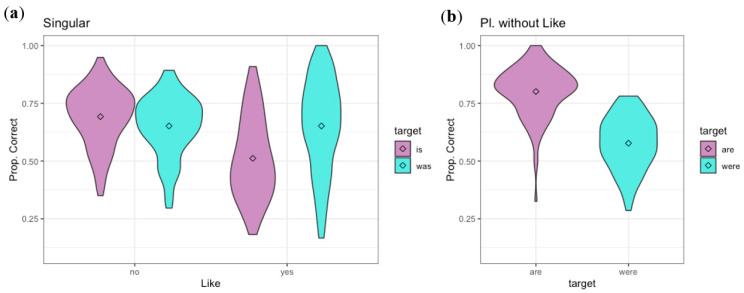
Results for auditory presentation of context with the target replaced by a beep sound (distribution of listeners’ averages over items). Dots indicate means for each condition. (**a**) “Is” and “was” targets. (**b**) “Are” and “were” targets.

The results for Experiment 2 (auditory, with beep replacing target) appear in Figure 3. The data were analyzed using the same designs as for Experiment 1, again with proportion correct as the dependent variable. As in Experiment 1, an interaction of the tense and “like” factors motivated examining the fixed factor of tense for each present–past pair separately (“is/was like,” “is/was” without “like,” and “are/were” without “like”). (By-subject ANOVAs for details of significant interactions, as in Experiment 1 above: Singular data: tense: F(1,110) = 5.05, *p* < 0.03, like: F(1,110) = 105.56, *p* < 0.001, interaction: F(1,110) = 53.17, *p* < 0.001.). The same methods for model selection and choice of random effects structure were used.(For *is/was without like* and *are/were without like*, the model was Correct ~ Tense + (1+Tense|Subject) + (1|Item); *is/was with like* used Correct ~ Tense + (1|Subject) + (1|Item). Models including speaker random intercepts gave either singular fit or failure to converge warnings.). For the “is/was like” items, the effect of tense was significant, with more accurate responses for “was” than “is” items (β = −0.63, z = −3.23, *p* < 0.005), while tense had no significant effect for “is/was” without “like” (β = 0.20, z = 0.92, *p* = 0.36). Detectability and bias measures appear in Table 1 above. For the “is/was like” items, listeners were somewhat biased toward the past tense response, but less so than in the orthographic task of Experiment 1. They showed greater detectability for the tense distinction if the following word was not “like.”.

For the “are/were” pair (without “like”) listeners showed significantly more accurate perception of “are” (present) than “were” (β = 1.24, z = 5.44, *p* < 0.001). This reflects a bias toward the present tense response, as well as some ability to hear the distinction (Table 1).

### 3.3. Discussion

These results show that listeners are able to extract some information about whether the verb is present or past tense from the surrounding syntactic and semantic information in the auditory modality, as well as the visual modality (Experiment 1 above). As with the visual modality, they find more information about verb tense in the sentential context if the following word is not “like.” Before “like,” listeners are biased toward the past tense option, although not as strongly when the information is presented auditorily as when it is presented orthographically. This suggests that listeners take the possibility of the historical present into account more when they can hear that the utterance comes from a casual conversation. That is, the tendency to assume that the verb must be in the past tense because it is reporting a past conversation is weaker if they hear the speech than if they see the written sentence.

Overall, the results of Experiment 2 confirm those of Experiment 1, but show a weaker bias toward past tense before “like” (more chance of taking historical present into account) in the auditory modality. Experiment 2 confirms that listeners are able to gather some information from the surrounding syntactic and semantic context about the verb tense when hearing the speech: across the four conditions without following “like,” listeners averaged 68.2% correct answers. Clearly, the surrounding syntactic and semantic context provides some information even when it is presented auditorily, a single time, at the fast rate of spontaneous speech, but it does not provide enough to fully disambiguate the verb tense, as 68.2% correct is far from 100%.

Experiments 1 and 2 provide two types of baseline that reveal how much information about the verb tense native speakers can obtain from the syntactic, semantic, and prosodic context around that verb. When we give conference talks or class presentations on reduced spontaneous speech, we find that a common informal assumption about how listeners succeed in understanding reduced conversational speech is that they do it by understanding the surrounding syntactic and semantic information, which may include some more clearly pronounced words, and making inferences based on it. Shockey [33], section 4.2.2 suggests that hearing several words of an utterance’s context after a reduction may sometimes allow listeners to suddenly recognize the reduced word, which could not be recognized until then. A large amount of past literature generally references context outside of the word as being helpful to the perception of reduced words and sounds, although not necessarily syntactic and semantic context, e.g., [4,5,6,7,8,9,15,17,22]. Experiments 1 and 2 show that there is useful information present in the rest of the sentence, but not enough for listeners to identify the verb tense very accurately. One might expect that native listeners would be more than 68–69% accurate in perceiving verb tense. Therefore, the acoustic signal within the target word/phrase itself may be providing considerable information, even when the speech is reduced. Experiment 3 addresses this issue.

## 4. Experiment 3: Auditory Targets with and without Context

In Experiment 3, we investigated how much information about the reduced speech of the verb native listeners can obtain from the acoustics of the target itself, with or without context. In some stimulus tokens, the portion of the signal corresponding to “you’re” consists only of a single central vowel (e.g., Figure 2), or the portion corresponding to “you were” when heard in isolation sounds like an excellent example of “you’re.” When hearing reduced tokens in isolation, it can be tempting to infer that listeners cannot possibly be using the small amount of acoustic information in the target word/phrase itself to perceive the content. However, listeners may be relying on the acoustic information even when it is very reduced, perhaps even if this acoustic information leads them to misperceive the word, for example, if a reduced token of “you were” is misperceived as “you’re” because it contains only one vowel. Thus, one question is how much use listeners make of the acoustic information within the target itself, even if it may lead to the wrong answer. We can answer this by presenting listeners with just the target word/phrase, in isolation.

Hearing the target word/phrase in isolation and hearing the rest of the sentence without the target (Experiment 2) are not simply two separable parts of perceiving the whole utterance. Coarticulation between the target word/phrase and the sounds just outside of it may be helpful to listeners. Furthermore, context provides information about the speech rate and speech style of the utterance, and listeners may use this to normalize their expectations about the duration of words, as shown in [12,15] and related work. If the surrounding speech is very fast, the boundary between what counts as “he’s” vs. “he was” may fall at shorter durations than if the surrounding speech is slow, because the listener expects the speech in the target word/phrase to be fast as well. If the information the context provides is about speech style rather than just rate, knowing that the surrounding speech is spontaneous and reduced could have the same effect of causing the listener to expect shorter, more reduced pronunciations of the longer possible parse “he was.”

In this experiment, we presented listeners with the target word/phrase and three levels of context (the same levels as in [5]), blocked by amount of context. This methodology is similar to that in [23]. In one condition, listeners hear only the target (e.g., “we’re,” “he was”), with no additional context (isolation condition). This condition thus provides listeners with whatever acoustic information occurs within the target word/phrase itself, but nothing more. In the limited context condition, listeners hear from the onset of the vowel preceding the target through the offset of the vowel following it (the stretch including the target and out to the edges of its surrounding vowels). For example, for the utterance “’Cuz he already told Steve he was in the wedding,” the listener hears whatever portion of the signal corresponds to the phoneme string /iv hi wʌz ɪ/ (“-eve he was i-”). Any consonants intervening between the nearest vowel and the target are also included in this condition (e.g., /v/ in “Steve”). The speech out to the edge of the surrounding vowels should be enough to give listeners some information about speech rate independently of the target itself, but not enough to allow them to recognize the neighboring words with certainty in most cases. When the following word is “like,” it is usually recognizable, since the vowel includes coarticulation with the following “k” and “like” is a very probable word after many of the targets. However, most other surrounding words cannot be recognized with certainty based on the limited context. For example, in the limited stimuli, the syllable after the target in both “I thought you were asking me” and “and we were outside the bookstore” sounds like “at” rather than “asking” or “outside.” We believe the limited level of context does provide some information about speech rate, because [34,35] show that listeners do not assume that they might be hearing only part of a segment when a segment is cut off; instead they parse whatever acoustic cues they have heard as a segment, so in this case listeners were unlikely to assume that the neighboring vowels could be longer than what they heard. Furthermore, the intervening consonants such as /v/ in “Steve” in this case also provide some speech rate information. This amount of context could be confusing to listeners, since it includes incomplete words, but it is important to test whether context is useful independent of lexical information.

The third level of context allows the listener to hear the entire utterance, including the target (full context condition). This is the same acoustic signal as in Experiment 2, but with the target presented as well, not obscured in any way. Thus, this condition provides all possible types of information that occur within the utterance: the acoustics of the target word/phrase itself, speech rate, the syntactic and semantic context, and cues to the speech style of the utterance. The only additional source of information this condition does not provide is the long-term discourse context: information about what the speaker has been discussing up to this point in the conversation, or long-term acoustic cues such as speech rate beyond the single utterance (which shows an effect as speech rate across the experiment in [36]). Thus, in this experiment, listeners can use the acoustic cues in the target word/phrase itself and can also use the information present in various amounts of surrounding context. This differs from Experiments 1 and 2, where only the context information was presented, without the target itself.

### 4.1. Methods

#### 4.1.1. Materials

The materials were the same recordings used in Experiment 2 except for the portion of the signal presented. For the full context condition, the stimuli were identical to those of Experiment 2 except that the target word/phrase was not replaced by a beep. These materials were simply extracted from the surrounding speech stream using the boundary criteria described for Experiment 2 and were not further manipulated. For the isolation condition, the same portion that was removed for Experiment 2 (the target word/phrase) formed the stimuli for the isolation condition of Experiment 3.

For the limited context condition, the portion from onset of the preceding vowel through the target word/phrase and up through the end of the following vowel was presented, e.g., for the utterance “you know, you were telling me about his roommate,” the stimulus was the portion of the signal corresponding to /o^w^ ju wɚ tɛ/. If a pause occurred in between the target and its nearest vowel, that was also included, as in this token, which had a short pause between “you know” and “you were.” In some stimuli, the target word/phrase was at the beginning or end of the full context utterance, as in “He was totally making fun of me today.” In such cases, the limited context stimulus included the neighboring vowel on the side that had one, e.g., /hi wʌz to^w^/.

The criteria for placing the boundary at the outer edge of the neighboring vowel were the same as described in Experiment 2 above, relying on onset/offset of F2, onset of voicing in the case of a voiceless obstruent followed by a vowel, the most sudden change in amplitude of formants for glide-vowel or vowel-glide boundaries, sudden change in the distribution of energy for nasal–vowel or vowel–nasal boundaries, etc. If the neighboring vowel was one of a string of vowels, as in “we were doing it” with the /uɪŋɪ/ portion of “doing it” realized only as a string of nasalized vowels, then the boundary was placed halfway through the F2 transition from the vowel adjacent to the target to the next vowel if there was an F2 transition (/uɪ/ in this case), and at the end of the vocalic stretch if the vowels were merged into a single vowel. If the consonant after the neighboring vowel was realized entirely as a creaky voice in place of a glottal stop (e.g., “they’re not recording” with the /t/ of “not” as creaky voice), the onset of the creaky voice was considered to be the boundary between the vowel and consonant. If a target’s neighboring vowel was absent, leaving a syllabic sonorant (e.g., neighboring “and” realized as [n̩]), then the end of the sonorant consonant was used as the end of the neighboring “vowel.” However, if a target’s neighboring vowel was absent and there was no sonorant consonant present, as in deletion of the vowel of “the,” then the limited context extended to the outer edge of the next vowel that was phonetically present. Since the word “the” sometimes has very little acoustic content at all, this is necessary to have a neighboring vowel present. All boundary points were adjusted to the nearest zero-crossing to avoid adding click artifacts.

#### 4.1.2. Participants

74 native speakers of American English who had not participated in the previous experiments participated. They were drawn from the same population as the participants for Experiments 1 and 2 and had similar language backgrounds to those participants. These participants also received extra credit in their Linguistics course for participation.

#### 4.1.3. Procedures

The procedures were the same as for Experiment 2 above, except that listeners received the three conditions (full context, limited context, isolation) in blocks, with a break between each condition. The conditions were presented in that order (from most to least information) for all listeners, because the full context condition is most similar to the material presented in Experiments 1 and 2. Thus, having listeners respond to the full context block first, so that they cannot be influenced by having heard the other context conditions, makes the full context results directly comparable with the results of Experiments 1 and 2. Furthermore, among the 184 items, most with targets such as “he’s, we’re, we were” etc., it is unlikely that listeners would be able to remember specific items and apply knowledge from having heard the full context condition when hearing the corresponding item in other conditions, especially since the other conditions did not present enough context for surrounding words to generally be recognizable.

Within each condition (full, limited, and isolation), listeners first heard five practice items made from the same tokens as were used for practice items in Experiment 2. Experimental items were blocked by speaker (within each condition block), as for Experiment 2, and as in that experiment one acclimation item by the same speaker was presented before the experimental items, but was not indicated to participants as being different from the experimental items. Data from acclimation items were not analyzed. In total, within each context block, listeners heard five practice items, 18 acclimation items, 184 test items, and 4 additional test items that were later excluded for comparability across experiments. While all listeners received the context blocks (full, limited, isolation) in the same order, within each context block, the order of the speaker’s voices and the order of items within each voice (after the acclimation item) was a different randomization for each listener.

The display on the computer monitor and the response alternatives for this experiment were identical to those in Experiment 2. Listeners were instructed to press the correct button to show whether the word/phrase within the sentence was, for example, “he’s/he is” or “he was.” None of the utterances contained the same target word/phrase twice, so there was no ambiguity as to which target was intended. The entire experiment took approximately 50 min. All other aspects of procedures were the same as Experiment 2. The time-out, after which the computer advanced to the next item if the listener failed to respond, was 9 s from onset of the stimulus. This occurred only on 320 trials, 0.8% of the data. These trials were excluded from further analysis. Median reaction time across all blocks was approximately 1200 ms from stimulus onset.

### 4.2. Results

**Figure 4:**
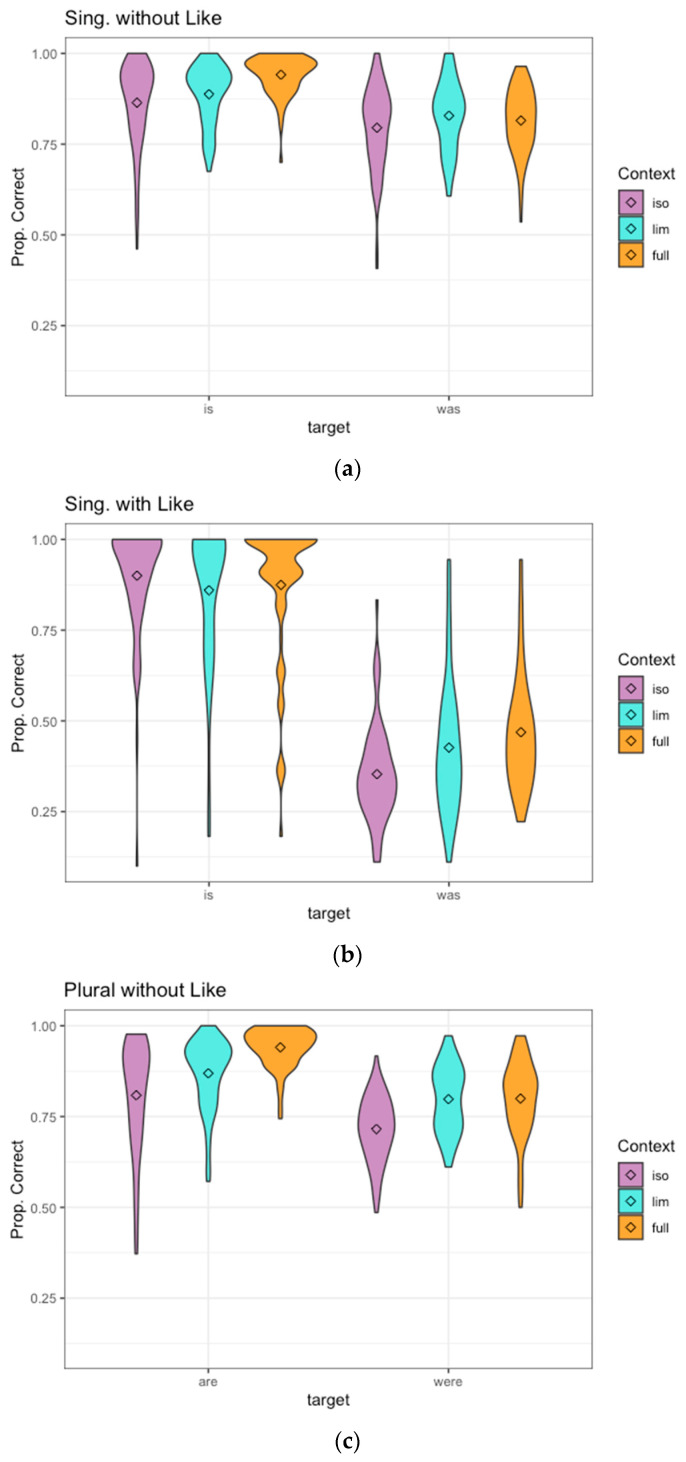
Results for listeners hearing targets with various amounts of context (iso = Isolation, lim = Limited, full = Full utterance context, distribution of listeners’ averages over items). Dots indicate means for each condition. (**a**) “Is” and “was” targets not followed by “like.” (**b**) “Is” and “was” targets followed by “like.” (**c**) “Are” and “were” targets not followed by “like.”.

The results for Experiment 3 appear in Figure 4. This experiment had context (isolation, limited, full) as an additional factor beyond those used in the experiments above, and context was the factor of primary interest, to answer which types of information the listener uses in perceiving potentially homophonous reduced speech forms. Limited context was used as the reference level for all analyses in order to reveal whether limited context allows listeners to perceive the target more accurately than the absence of context does, and whether they are able to extract more information from the full context than the limited. Initial models showed significant interactions between context and the other factors (tense and presence/absence of “like”), which motivated testing just the context factor for six subsets of data (“is like”, “was like”, “is” without “like”, “was” without “like”, “are” without “like”, “were” without “like”). (By-subject ANOVAs for details of significant interactions, as in Experiment 1 above: Singular data: tense: F(1,73) = 163.81, like: F(1,73) = 2141.84, context: F(2,146) = 18.57, tense x like: F(1,73) = 257.14, tense x context: F(2,146) = 5.37, *p* < 0.01, like x context: F(2,146) = 1.07, *p* > 0.10, tense x like x context: F(2,146) = 15.35, all *p*’s < 0.001 unless otherwise specified.). Model selection and choice of random effects structure was done in the same way as for Experiments 1 and 2. (The model for *is without like*: Correct ~ Context + (1|Subject) + (1+Context|Item); *was and are without like*: Correct ~ Context + (1+Context|Subject) + (1|Item) + (1|Speaker); *is with like*: Correct ~ Context + (1|Subject) + (1|Item); was with like: Correct ~ Context + (1+Context|Subject) + (1+Context|Item); *were without like*: Correct ~ Context + (1+Context|Subject) + (1|Item).). We predicted before beginning the analysis that the perception of “are” vs. “were” would be very different from the perception of “is” vs. “was”, because of the segmental content of the words, and hence the acoustic cues, are so different. Therefore, the data cannot be analyzed when pooled over the singular and plural verbs, as was confirmed by the significant interactions with context. The “like” factor was added post hoc, as discussed for Experiment 1 above, because it had a large interaction with other factors, making it impossible to conduct a meaningful single analysis over items with and without “like.”

For the singular “is” targets not followed by “like,” the limited context was perceived significantly better than the tokens in isolation (β = −0.55, z = −3.52, *p* < 0.001), and full context was perceived better than the limited context (β = 0.74, z = 4.33, *p* < 0.001). For a singular “was” not followed by “like,” isolation was perceived less accurately than limited context (β = −0.29, z = −2.42, *p* < 0.02), but full context provided no significant additional benefit (β =−0.17, z = −1.54, *p* = 0.124). Thus, for “is/was” targets not followed by “like,” both types of context facilitated the perception of “is” but only limited context helps listeners recognize “was.” Hearing the content of the rest of the utterance (full context) did not provide any additional benefit when listeners are hearing “was.” The implications of this for listeners’ use of various types of information will be discussed in Section 4.3 and Section 5 below.

For the “is” targets followed by “like,” listeners performed significantly better in isolation than with limited context (β = 0.59, z = 3.56, *p* < 0.001). This apparent negative effect of limited context on perception will be discussed below. Full context led to no difference relative to limited context (β = 0.26, z = 1.67, *p* = 0.094). For “was” targets followed by “like,” just as for “was” without “like” above, limited context was perceived significantly better than isolation (β = −0.57, z = −3.16, *p* < 0.005), but full context provided no additional benefit (β =0.29, z = 1.41, *p* = 0.159).

Turning to the plural “are/were” pair, of which only those without “like” are analyzed as noted above, the “are” targets showed significant improvement in perception with each additional level of context (limited vs. isolation: β =−0.39, z = −4.00, *p* < 0.001; full vs. limited: β =0.94, z = 7.59, *p* < 0.001). For the “were” targets, limited context led to improved perception relative to isolation (β =-0.62, z = −7.34, *p* < 0.001), but Full context provided no additional benefit (β =0.05, z = 0.54, *p* = 0.588). This is the same pattern as for the “is/was” pair without “like”: both types of context improved the perception of the shorter present tense form, while only limited context improved the perception of the longer past form. The longer past tense forms “was” and “were” (including “was” both with and without “like”) showed no additional benefit when listeners hear the entire surrounding utterance in full context.

Detectability and bias measures appear in Table 1 above. The detectability results show that listeners are able to distinguish present and past tense verbs much better if the stimulus sentence was not produced with a following “like” (whether that following word is presented or not). The bias results in Table 1 for this experiment indicate that the listeners are biased toward the shorter present tense response in all conditions, unlike in Experiments 1 and 2.

### 4.3. Discussion

The results of Experiment 3 show that when potentially reduced function words are not followed by “like,” listeners are able to extract considerable information about the intended function word from the acoustics of the target word/phrase itself. The relatively high accuracy for “is/was” and “are/were” without following “like” in the isolation condition (average of 83 and 76% correct, respectively) provide evidence of this. The availability of context, whether the speech rate and coarticulation context afforded by the limited condition or the syntactic, semantic, and prosodic context contained in the full condition, does lead to better perception. However, this improvement is somewhat modest, with accuracy improving only to 88% (“is/was” without “like”) and 87% (“are/were” without “like”) in full context. Information in the surrounding utterance is not enough to fully disambiguate the target words, even with the combination of bottom-up and top-down processing that contextual information allows. The acoustic information in the target word/phrase itself seems to contribute more than either type of context.

With the data in this experiment, we could examine closely which types of context contribute to listeners’ perception. For all of the past tense target conditions (“was” with “like,” “was” without “like,” “were” without “like”), limited context improves accuracy relative to isolation, but full context leads to no significant further improvement. The past tense targets are always the longer linguistic form in the number of phonemes relative to their present tense counterpart (e.g., “was” vs. “is/’s”), and in careful speech, the past forms must constitute a syllable, whereas the present forms can be contracted to a single consonant. The results indicate that when listeners hear a reduced production of a longer past form that sounds ambiguous or sounds like the corresponding shorter present form, the information in the limited context helps them to reconstruct the longer form from the reduced acoustics, but the semantic and syntactic information of the rest of the sentence does not help them with this aspect of processing. The limited context condition, extending only to the outer edges of the nearest vowels to the target, provides listeners with information about the speech rate of the utterance and coarticulation with neighboring sounds, but is not enough to provide consistent or accurate syntactic or semantic information. When the following word is “like,” it is recognizable in limited context. Other surrounding words are not consistently recognizable from the limited context stimuli, and may be misperceived as other words or not recognized as words, although some of the neighboring words may be correctly perceived as well. It seems that when listeners hear a reduced form in spontaneous speech, part of the process of perceiving it is evaluating it relative to the speech rate of the surrounding speech, as also shown in [5,15]. If the surrounding speech is fast, then a given duration might be too long to be a good candidate for “we’re,” but would be a better candidate for “we were.” If the listener does not have access to information about the surrounding speech rate, as in the isolation condition, they might assume the same production was “we’re” instead. This is similar to Miller & Volaitis’ [12] finding that listeners adjust their category boundaries for aspirated vs. unaspirated stops depending on the surrounding speech rate. Here, listeners are adjusting their category boundary for “we’re” vs. “we were” or “he’s” vs. “he was” when they have information about the surrounding speech rate.

It is possible that the limited context supplies perceptual information other than speech rate, for example simply because including the target’s neighboring segments provide information through coarticulation. That is, there could be additional perceptual cues to the segments of the target word/phrase in the adjacent segments. For example, in /iv hi wʌz ɪ/ extracted from “told Steve he was in the wedding,” it is possible that the final /ɪ/ could contain perceptual cues to the preceding word “was.” However, it is unlikely that coarticulation rather than speech rate is the primary source of perceptual improvement in the limited condition. For all target words/phrases, the past and present target forms begin and end with the same segments (e.g., “he’s” and “he was” both begin with /hi/ and end with /z/). Coarticulation between the final /z/ of the target and its following vowel may make the /z/ more perceptible, but this would not help listeners distinguish “he’s” from “he was.” It is more likely that the crucial information in the limited condition is speech rate. When listeners hear the fast surrounding speech, they realize that the duration of the “was/were” targets is too long to be the shorter present tense form at that speech rate. This allows them to hypothesize that segments have been deleted and reconstruct the longer past tense form.

For the shorter present tense targets, the presence/absence of a following “like” affects which type of context improves perception. For both “is” and “are” without “like,” each type of context leads to significant improvement. Listeners are already biased toward the shorter present tense responses even in isolation in these conditions. Having either speech rate or syntactic and semantic information available further strengthens their judgement that these forms are the present tense option. For “is” followed by “like,” the only effect of context is to reduce the accuracy of perception rather than improve it, only with the addition of limited context. This unexpected negative effect may reflect context, leading listeners to rely less on bias (which is strongly toward “is” in this condition). That is, proportion correct dropped not because the listeners became worse at realizing they have heard “is,” but rather because limited context gives them enough information to rely less on bias toward “is” and more on the ambiguous cues they hear. This direction of bias toward the “is” response before “like” is notably different from the bias in the “is/was like” conditions of Experiments 1 and 2, as will be discussed below.

This bias toward “is” in utterances with “like” was present even in the isolation condition, where listeners cannot hear the “like.” (We verified by listening that coarticulation during the verb is not sufficient to perceive that “like” follows.) To verify statistically (beyond the bias value toward “is” in Table 1) that responses are quite different if the stimulus was extracted from before “like” than if it was not, we performed a post hoc comparison of “was” in isolation in the “like” vs. non-“like” conditions. The proportion correct is significantly lower in stimuli where a “like” (unheard by the listener) had originally followed the “was” (β =−3.18, z = −5.20, *p* < 0.001). The fact that this bias toward “is” occurs even when the listeners do not hear the “like” (isolation) suggests that the collocation with “like” causes a difference in the acoustics of the preceding target word itself, which is what influences listeners’ behavior. For speakers who use quotative or discourse adverb “like,” phrases such as “he was like,” “he’s like,” “I was like” are extremely common [28,30], and thus especially subject to reduction [37,38,39,40]. Since reduction makes forms shorter and makes the /w/ of “was” or “were” less distinct [4,25,41], more reduced productions of “he was” will sound more like “he’s.” Thus, speakers reduce the entire phrase pronoun-is/was-like because of its high frequency, and this gives the “is/was” before “like” perceptual cues expected for “is” rather than “was.” This leads to listeners’ strong preference for the present tense “is” response for stimuli before “like” in all context conditions of Experiment 3. The bias becomes somewhat weaker in limited and full context conditions as listeners gain more ability to detect the difference between “is like” and “was like.”

## 5. General Discussion

These three experiments test what types of information listeners use when perceiving reduced, potentially homophonous function words such as “he’s” vs. “he was” in spontaneous, conversational speech. The types of information available include the acoustic information present in the target words themselves, the rate of surrounding speech, coarticulation with nearby sounds, cues that inform the listener that the speech style is conversational and reduced, and syntactic and semantic cues in the rest of the utterance, such as tense of other verbs or presence of a time adverb like “yesterday.” Across four comparisons enumerated below, the current results suggest that listeners make more use of acoustic cues than of anything else, while using both bottom-up and top-down processing to reach a percept. This finding of dominance of acoustic cues over meaning is consistent with findings of [6,22].

First, comparison of Experiments 1 and 2 to the isolation condition of Experiment 3 shows that listeners are able to extract more information about “is” or “was” and “are” or “were” from the brief, reduced acoustic cues in the target word/phrase itself than from the entire surrounding context, no matter whether it is presented auditorily or in writing. (Experiment 2 provided listeners with acoustic cues to the context, but not to the target itself.) For this, we examined the conditions without “like,” to avoid the other factors influencing those utterances. The average proportion correct and the d’ measure of detectability in Table 1 were considerably higher for the isolation condition of Experiment 3 than for either Experiments 1 or 2, both for “is/was” and “are/were” without “like.” To confirm this statistically beyond the d’ values, we calculated each listener’s average proportion correct for each word pair (is/was vs. are/were without “like,” averaged over items first since number of present and past items is not equal), and used a linear mixed effects analysis to confirm that proportion correct was significantly lower in each of Experiments 1 and 2 than in Experiment 3′s isolation condition. (CorrectNum ~ Exper * Wordpair + (1|Subject); the interaction of Wordpair by Experiment was significant, so the Wordpair was releveled to test with both *is/was* and *are/were* as the reference level. With *is/was* as reference level, Exper. 1 shows significantly lower proportion correct than Exper. 3 Isolation (set to reference level): β = −0.15, t = −9.25, *p* < 0.001; Exper. 2 also does: β = −0.16, t = −12.56, *p* < 0.001. The same is true with *are/were* as reference level: Exper. 1: β = −0.06, t = −3.91, *p* < 0.001, Exper. 2: β = −0.07, t = −5.10, *p* < 0.001.). Examining the average proportion correct for a present/past pair avoids the issue of bias in either direction in order to focus on how well listeners can hear the difference. As discussed in the Methods section of Experiment 2, the average duration of the isolation stimuli was only 262 ms, and many were severely reduced, as in Figure 2. Out of context, in isolation, these words/phrases can be very difficult to perceive. However, listeners perceived the targets more accurately from just the acoustic information in the target itself than they did when they were presented with all the context information in the utterance, including semantic and syntactic cues such as time adverbs, even if the context is presented in writing with ample time to read it several times. These results suggest that listeners can gain more information about reduced function words in conversation from the acoustics of just the word itself than from the entire total of all information in the context.

Second, in all three past tense conditions of Experiment 3 (“was” without “like,” “was like,” and “were” without “like”), the only type of context that improved listeners’ accuracy significantly was the limited context, as compared to the absence of context (isolation condition). In all three cases, the full context condition led to no significant additional improvement. The limited context is not enough to allow the accurate perception of most words adjacent to the target except for the word “like.” The limited context extends only as far as the outer edge of the neighboring vowels, as in /iv hi wʌz ɪ/ (“-eve he was i-”) from “Cuz he already told Steve he was in the wedding.” Because listeners often could not recognize the context in the limited condition as words, the addition of limited context might make the stimuli somewhat confusing relative to the isolation condition. Still, listeners were able to use the limited context to help them partially recover from the reduction of the was/were forms and to correctly parse them as the longer past tense (was/were) forms. Hearing the entire utterance did not provide significant additional benefit.

As discussed above in Experiment 3, the most likely explanation for what information listeners use from the limited context is speech rate information. Hearing that the surrounding speech is fast could shift the listeners’ boundary between “he’s” vs. “he was” to a shorter duration, because in fast speech, the longer past form “he was” is expected to take less time than in slower speech. Thus, listeners can use the speech rate information (or perhaps information about speech style and degree of reduction) in the limited context to help them recover the longer form from its reduced, shorter pronunciation. This is similar to how a listener adjusts the range of expected values for VOT depending on surrounding speech rate [13,17]. It is also similar to the finding [15] that the surrounding speech rate influences listeners’ perception of whether function words such as “or” are present at all (“leisure or time”/“leisure time”) and to the finding [36] that listeners also use long-term speech rate over the context of the experiment for this type of normalization. Notably, the addition of syntactic and semantic information from the full context did not help them significantly more beyond the limited context in the perception of the past tense conditions. This also suggests that acoustic cues (in this case speech rate and/or style) are more important than the larger context of meaning. Still, it is likely that listeners are combining bottom-up and top-down processing to perceive speech, even when the acoustic cues dominate the percept.

Third, the direction of bias in the “like” conditions provides another argument that acoustic cues outweigh other cues. In Experiment 1, where no acoustic cues were available and participants received only syntactic and semantic information, the “is/was like” conditions showed a strong bias toward “was.” This is evident from the positive β value in Table 1 and the high proportion correct for “was like” stimuli and significantly lower proportion correct for “is like” stimuli. (If the participants decided entirely based on bias toward “was,” with no detectability, we would see 100% correct for “was” and 0% correct for “is.”) In Experiment 2, where the same context information was presented auditorily, the bias is in the same direction, but is not as strong. Experiments 1 and 2 suggest that when participants have only the syntactic and semantic information available, they assume that these sentences containing “like” are likely to be in the past tense because they are often reporting speech that happened in the past or describing a past situation. Given only the syntactic and semantic context, listeners do not take the possibility of historical present usage into account well, and instead assume past events have past tense verbs. The reason that the bias toward “was” before “like” is stronger in Experiment 1 than 2 may be that seeing the written form leads participants toward a more prescriptive judgment of which verb tense would be “correct” in a sentence.

However, in Experiment 3, where listeners can hear the target word/phrase itself, the direction of bias in the “like” conditions reverses, to a rather strong bias toward “is.” This is true even in the isolation condition, where the listeners could not hear the “like.” It is also true in the full context condition, which differs from Experiment 2 only in that the target word/phrase itself was also played. Thus, the acoustic information in that target word/phrase is sufficient to override all of the syntactic and semantic information that is available in the utterance context (the same information available in Experiment 2) and reverse the direction of bias. Both the target word and the utterance context cause a bias, but in opposite directions. If both are available (full condition of Experiment 3), the acoustic cues of the target word override the syntactic and semantic information of the context.

Fourth, the direction of bias in all conditions of Experiment 3 (with and without “like”) is toward the present response (“is” or “are”, reflected in negative β values for all conditions). Acoustic information in the stimulus target words themselves can be a source of bias. Because the stimuli are from spontaneous, casual conversations, many of the productions of target words are rather reduced, and reduction makes the past tense forms “was, were” sound more like “is, are,” by making them shorter with less distinct segments. Thus, if listeners rely strongly on the acoustic cues in the targets themselves, and if many tokens contain reduction, we would expect to see bias toward the present tense responses. The acoustic cues listeners rely on may mislead them into choosing the present tense response more often than it was actually produced. While in much research bias is something undesirable to be removed in the analysis, in this case, it provides evidence for listeners’ use of the acoustic cues in the target words/phrases, since reduction shifts these acoustic cues toward the present tense end of the distinction. Listeners favor the acoustic information in the targets over other information even when it misleads them.

Our findings that acoustic cues outweigh syntactic and semantic cues in the utterance context relate to the findings [21,42] that listeners make less use of semantic information in reduced pronunciations of words when recognizing subsequent words, and that they need more time to process reduced speech before they can use the semantic information in a reduced word. Drijvers et al. [24] found that reduction makes it more difficult for listeners to activate semantic information. Acoustics also outweigh word bigram probabilities in context as more acoustic information becomes available in [23], but in that case, words did not have homophones. In the current potentially homophonous short phrases such as “he’s/he was” and “we’re/we were,” we found that the acoustics outweigh the meaning of the utterance context.

## 6. Conclusions

Overall, these results show that native listeners of English integrate several types of information during the process of perceiving function words in reduced spontaneous speech. They use the acoustic information within a word itself, the surrounding speech rate, and syntactic and semantic information from the rest of the utterance, as well as potentially other types of information not tested here. For example, we sometimes found that it is easier to understand a highly reduced utterance if one has heard the preceding conversation and knows what topics the speakers are discussing, but we were unable to test this here.

The listeners showed a stronger reliance on acoustic cues than on any other type of information. This does not mean that listeners fail entirely to use syntactic and semantic information in the utterance context: the significant improvement from limited to full context for “is” and “are” without “like” in Experiment 3 show use of that information. However, four comparisons across various parts of the three experiments all lead to the conclusion that acoustic cues dominate: (1) participants perceived the targets “is,” “are,” “was,” “were” more accurately based on just the very short recording of the target phrase in isolation than they did based on the entire utterance context; (2) acoustic information in the immediately surrounding few sounds (limited context) helps listeners to recover from reduction to recognize the longer past tense forms was/were, while the addition of the entire meaning of the rest of the utterance does not provide any further benefit; (3) for “is like/was like,” the utterance context biases participants toward the “was” response, but just the addition of the acoustic cues in the target is sufficient to reverse that to a bias toward “is,” even when the utterance context is also heard; (4) whenever listeners hear the acoustics of the target, they show bias toward the shorter “is” or “are” response, consistent with following the acoustic cues of this reduced speech. This dominance of acoustic cues is especially interesting because it contradicts one potential explanation for how listeners understand reduced speech: that other words in the utterance are pronounced more clearly, and listeners use those instead to avoid having to parse the reductions. Instead, what we find is that listeners favor whatever acoustic information is available.

## Figures and Tables

**Table 1:** Signal detection measures d’ (detectability) and β (bias), and average proportion correct across the present and past verb of the pair, for the tense distinction for each pair of conditions. Positive β indicates bias toward the past response, negative toward the present response. All experiments are included here for ease of comparison. Number of items in each condition appears in Appendix A (Table A1).

Experiment/Condition	Context	d’	β	Avg. Prop. Correct
Exper. 1 (Orthography)				
is/was, no “like”		1.005	0.054	0.692
is/was, with “like”		0.504	0.306	0.583
are/were, no “like”		1.062	−0.052	0.702
Exper. 2 (Auditory, target replaced by beep)				
is/was, no “like”		0.889	−0.048	0.672
is/was, with “like”		0.414	0.078	0.581
are/were, no “like”		1.042	−0.338	0.690
Exper. 3 (Target plus various contexts)				
is/was, no “like”	Isolation	1.927	−0.269	0.830
	Limited	2.162	−0.292	0.858
	Full	2.460	−0.820	0.878
is/was, with “like”	Isolation	0.910	−0.757	0.627
	Limited	0.852	−0.520	0.639
	Full	1.068	−0.653	0.672
are/were, no “like”	Isolation	1.443	−0.226	0.762
	Limited	1.943	−0.301	0.832
	Full	2.405	−0.868	0.871

## Data Availability

The human subjects permission and approved consent form included a statement that data would only be available to the researchers and their assistants or others collaborating with them. If you wish to collaborate on a future project involving the data, please contact the corresponding author.

## Appendix A

**Table A1:** Examples of the 184 target items used in all of the experiments, by condition. The target word is underlined, and the phrase or sentence given here is what was used for the “full” context.

Present Tense target verbs	Past Tense target verbs
“Is” without “like” (*n* = 40)	“Was” without “like” (*n* = 30)
He’s pretty closed off to everybody. But he’s still s- you know. It’s not like she’s gonna be there all day, you know? But, either way there’s I mean, I can’t… Grammy’s a grown, a grown woman.	Did you think he was ugly? She was really hyper earlier. No, it was probably last Tuesday. The other night he was at, um, like, his fraternity house. He was totally making fun of me today.
“Is” with “like” (*n* = 11)	“Was” with “like” (*n* = 16)
And she’s like, “Yay! I’m so excited for you!” Yes, he’s like superhyper. She’s like, “No! No more laptops!” So, I don’t, she’s like, she has an older computer at home. Maybe my Dad could help you, he’s like…	He was like… And he was like, “What’s wrong?!” I called Dad and asked him about the internet, and like, he was like… ‘Cuz she was like, “I’m gonna, you know, be screwed if I don’t.” It was like, the words of a giant.
“Are” without “like” (*n* = 43)	“Were” without “like” (*n* = 35)
Oh, you’re going to, uh, Gymboree with Sam? And then they, like, read it to see how good of a writer you are too, so… I don’t even know what we’re gonna do. So they’re gonna have like a random roommate. He’s like, “You are very lucky, ‘cuz that’s not how it works.”	You know, you were telling me about his roommate. We were gonna go out to dinner so he could see her, I dunno. He said they were dating. That was, my parents were so happy when I didn’t get a bid to a sorority. Plans got changed, ‘cuz like we were supposed to leave Thursday.
“Are” with “like” (excluded, *n* = 2)	“Were” with “like” (excluded, *n* = 7)
I was like, “What are you guys doing,” and they’re like… But, they’re like, “It’s cheaper that way!”	They were like, “Oh my God,” that’s, it’s like almost… Um, Kaibab, but they were like talking to a girl and there were ambulances there and stuff. So he was so funny, you were like… Yeah, when I talked to you, you sounded like you were like dying. But now it’s so far past Easter that we were like…
